# Cortically Dependent Motor Training Does Not Induce Abnormal Movements in DYT1‐Knock In Mice

**DOI:** 10.1002/brb3.71176

**Published:** 2025-12-31

**Authors:** Alexander T. Hodge, Mohammed A. Rasheed, Tiffany Lin, Christian R. Burgess, Daniel K. Leventhal

**Affiliations:** ^1^ Department of Neurology University of Michigan Ann Arbor Michigan USA; ^2^ Michigan Neuroscience Institute University of Michigan Ann Arbor Michigan USA; ^3^ Department of Molecular and Integrative Physiology University of Michigan Ann Arbor Michigan USA; ^4^ Parkinson's Disease Foundation Research Center of Excellence University of Michigan Ann Arbor Michigan USA; ^5^ Department of Biomedical Engineering University of Michigan Ann Arbor Michigan USA; ^6^ Department of Neurology VA Ann Arbor Health System Ann Arbor Michigan USA

## Abstract

**Purpose:**

DYT1 dystonia is the most common inherited dystonia, but mouse models recapitulating the human genotype do not exhibit overtly dystonic movements. Because cortical and striatal plasticity are implicated in dystonia pathogenesis, we hypothesized that repetitive performance of a cortically‐dependent reach‐to‐grasp task would induce abnormal dystonia‐like movements in DYT1‐knock in (DYT‐KI) mice. The goal of these experiments was to test that hypothesis.

**Methods:**

TorsinA ΔE (DYT1‐KI) mutant mice and non‐transgenic littermates (control) were trained to perform a cortically‐dependent single pellet reach‐to‐grasp task using an automated skilled reach‐to‐grasp apparatus. Task performance and the presence of abnormal movements were manually scored by reviewers blinded to genotype.

**Results:**

Six DYT1‐KI and five littermate control mice performed at least 500 skilled reaches per animal. There were no differences in success or fumble rate between DYT1‐KI and control mice. DYT1‐KI mice exhibited subtle abnormal limb shaking in five trials, but a similar movement occurred in one control mouse in one trial.

**Conclusions:**

DYT1‐KI mice learn and perform skilled reach‐to‐grasp comparably to control mice without developing meaningful abnormal movements.

## Graphical abstract

DYT1 dystonia is a familial autosomal dominant, childhood onset dystonia. Mouse models recapitulating the human genotype do not exhibit overtly dystonic movements. It is unclear whether cortically‐dependent tasks are necessary for the development of dystonic movements. DYT1‐KI and control mice perform a cortically‐dependent reach‐to‐grasp task comparably without developing abnormal movements.

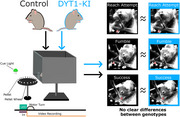

## 1 Introduction

Dystonia is a potentially disabling movement disorder characterized by abnormal, sustained, involuntary twisting movements. It can occur as a symptom of other conditions (e.g., Parkinson's disease, drug‐induced) or as a primary disorder. DYT1 dystonia is a familial autosomal dominant, childhood onset, usually generalized dystonia caused by a 3‐nucleotide deletion in the torsinA gene with approximately 30% penetrance (Ozelius et al. 1999 ; Ozelius et al. 1997 ). This low penetrance indicates that other factors (“second hits”) are necessary for the development of symptoms. Whether these factors are environmental, genetic, or both remains unclear.

These “second hits” are hypothesized to range from additional injuries to repetition of specific movements (Rauschenberger et al. 2021; Reinhold et al. 2024). Supporting repetitive movement as a “second hit,” overtrained actions can lead to focal dystonias like musician's dystonia and writer's cramp (Byl et al. 1996; Furuya and Hanakawa 2016; Altenmüller and Jabusch 2010). While torsinA mutations are most associated with generalized early‐onset dystonia, they are also rarely observed in patients with adult‐onset focal dystonia (Gasser et al. 1998; Grundmann et al. 2003; Magrinelli et al. 2018; Opal et al. 2002).

The presumed mechanism of overtraining‐induced dystonia is maladaptive plasticity in cortical and striatal circuits. Patients with a manifesting DYT1 mutation or idiopathic cervical dystonia have prolonged changes in motor cortical excitability under a repetitive transcranial magnetic stimulation protocol (Huang et al. 2010). Paired associative stimulation (PAS) experiments have shown increased excitability in patients with focal dystonia, though it is unclear if this is induced by LTP or LTD (Belvisi et al. 2013; Edwards et al. 2006; Siebner et al. 1999). In mouse models of DYT1 dystonia, abnormalities of striatal acetylcholine‐dopamine interactions and corticostriatal plasticity have been observed (Yokoi et al. 2021; Downs et al. 2022; Balcioglu et al. 2007; Sciamanna et al. 2012; Richter et al. 2019; Scarduzio et al. 2017; Martella et al. 2009). Despite these physiologic changes, mouse lines hemizygous for the DYT1 mutation have relatively minor motor abnormalities (Zhao et al. 2008; Dang et al. 2005; Song et al. 2012; Sharma et al. 2005) and do not exhibit the overt sustained agonist–antagonist cocontractions and abnormal postures that characterize human DYT1 dystonia.

One possible explanation for this dissociation between physiologic and motor abnormalities is that common murine behavioral assays do not require motor cortical control. Innate behaviors (e.g., grooming) and patterned lever pressing tasks can be performed without cortical input with minimal performance effects, at least after training (Berridge and Whishaw 1992 ; Hwang et al. 2019 ). On the other hand, mouse reach‐to‐grasp relies strongly on the motor cortex (Guo et al. 2015 ; Alaverdashvili and Whishaw 2008; Whishaw et al. 1991; Whishaw et al. 1993; Morendell and Huber 2017; Farr and Whishaw 2002). Dlx‐CKO mice that lack torsinA in forebrain GABAergic and cholinergic neurons are impaired in skilled reach‐to‐grasp with otherwise relatively preserved motor function (Kernodle et al. 2022 ; Pappas et al. 2015; Berryman et al. 2023). However, the relevance of the Dlx‐CKO model to human DYT1 dystonia is less direct. It remains unknown whether DYT1‐KI mice (which recapitulate the human genotype) are also impaired in skilled reach‐to‐grasp, and whether repetition of a cortically‐dependent skill can induce dystonia‐like movements in these mice. Such a finding would greatly improve the model's face validity and provide a platform for testing therapeutics.

To address this gap, we trained DYT1‐KI mice and non‐transgenic littermates (controls) to repeatedly perform skilled reach‐to‐grasp. We found no difference in reach accuracy, subaction timing, or sequencing between DYT1‐KI and control mice. There was also no significant difference in the prevalence of abnormal behaviors between DYT1‐KI and control mice. We conclude that repetition of a motor cortex‐dependent behavior in adult DYT1‐KI mice is insufficient to induce dystonia‐like movements. Whether a similar stressor can induce dystonia in human DYT1 carriers during a critical developmental period remains unclear.

## 2 Materials and Methods

2

### 2.1 Animals

All animal care and experimental procedures were approved by the University of Michigan Institutional Animal Care and Use Committee. Adult (≥ p60) DYT1‐KI mice harboring heterozygous mutations in the native mouse torsinA gene (six mice, two male/four female, Heterozygous Tor1A^+/ΔGAG^, mean starting age p73.49 ± 6.95 SD) and littermate controls (Tor1A^+/+^) of both sexes (controls, five mice, three male/two female, mean starting age p110 ± 60.25 SD) were housed at 22–24°C with a 12 h light:12 h dark cycle with water provided ad libitum (Goodchild et al. 2005). Experiments were performed in both light and dark cycles, with mice training once during the light cycle and once during the dark cycle each day. All mice were food restricted 3 days before pretraining. Food restriction was maintained for 6 days per week during training, with one day of ad libitum food access per week.

### 2.2 Reach‐to‐Grasp Task

#### 2.2.1 Task Design

**FIGURE 1:**
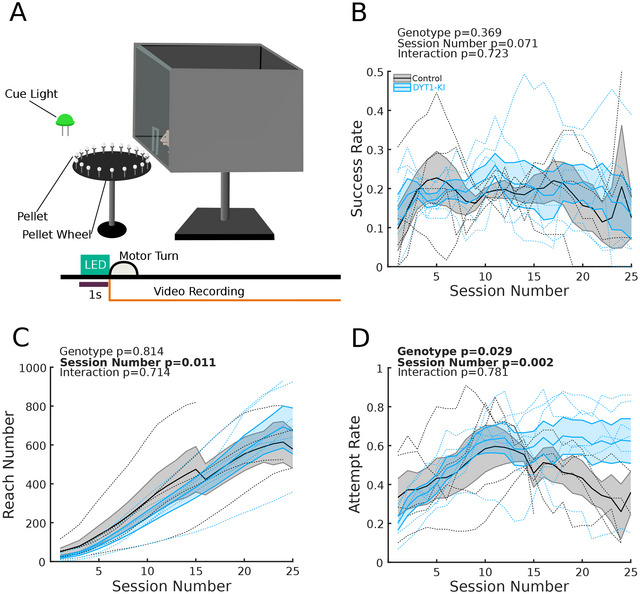
DYT1‐KI and control mice perform similarly in the skilled reach‐to‐grasp. (A) Schematic of automated reaching chamber (top) and task structure (bottom). Trials lasted 8 s and were separated by 30‐second inter‐trial intervals. (B) Mean success rate as a function of session number. DYT1‐KI mice and control mice were similarly successful throughout training. (C) Mean cumulative reach number as a function of session number. DYT1‐KI mice did not produce more reaches per session than control mice. (D) Mean attempt rates as a function of session number. While there is a difference in attempt rates between DYT1‐KI and control mice, the interaction of genotype and session number was not significant. For all plots, shaded areas are ±SEM, DYT1‐KI mice (*n* = 6) are in blue, controls (*n* = 5) in black. Dotted lines represent individual mice. Statistics in bold indicate *p* < 0.05. All mice performed at least 25 sessions, which are shown here. Full data for all 40 sessions are in Figure S1. Full statistics are listed in the supplemental table.

Skilled reach‐to‐grasp sessions consisted of 20 trials. Trials began automatically with presentation of a visual cue (green LED) for 1 s (Figure 1A). This was followed by the rotation of the wheel to present a new pellet in front of the reaching slot. Pellets were balanced on 20 pillars (18 mm length × 3 mm diameter machine screws). High‐speed videos (167 fps, Arducam OV9281 UC‐788 Rev.B with an M12 lens connected to a Raspberry pi 4b) were recorded for each trial, starting at cue presentation and lasting 8 s. Each recording was followed by a 30 s inter‐trial interval. Reaches that occurred between video recordings were not counted. Mice were trained until one of the three criteria was met: they produced 1000 total reaches (3 DYT1‐KI mice, 1 control mouse), trained for 40 sessions (3 DYT1‐KI mice, two control mice), or ceased reaching for three consecutive sessions (0 DYT1‐KI mice, two control mice). Mice were excluded from analyses if they produced fewer than 500 reaches (4 DYT1 mice and four control mice).

#### 2.2.2 Training

Pre‐training consisted of placing individual mice into a skilled reach‐to‐grasp chamber (Figure 1A) with a slot cut in the center of a clear plexiglass wall. Sucrose pellets were placed in this central slot to encourage investigation and associate the slot with a reward. Pellets were manually replaced by the experimenter after pellets were consumed or knocked out of the reaching chamber. Pellets were progressively placed further from the slot until mice consistently reached for pellets with fully extended forelimbs for two consecutive sessions. Mice took three to six pretraining sessions to achieve this milestone. “Training” refers to recorded sessions in which mice could reach for pellets from the pellet wheel (Figure S1A).

Training occurred once per day, 5 days per week. Some mice were trained a second time at least 4 h after their first sessions when possible. This consisted of 22 days (44 doubled‐up sessions out of 197 sessions total, 22%) for control mice and 51 days (102 doubled‐up sessions out of 276 sessions total, 37%) for DYT1‐KI mice.

Mice were placed into a reaching rig (Figure 1A). The reaching rig consisted of an elevated platform that held the reaching chamber, a motorized pellet wheel (holding 20 sucrose pellets), an infrared floodlight, and two Arducam cameras. A new pellet was presented at the beginning of each trial. Up to two rigs were active simultaneously in a 52 cm × 59.4 cm × 37 cm (internal dimensions) MedAssociates cabinet. Rigs were placed side by side 15 cm apart. Trials were simultaneously initiated for both rigs using a single Arduino. The chambers were illuminated by infrared lights but were otherwise in the dark. Three of the four reaching chamber walls were opaque. While mice were unable to see one another, it was feasible that mice could hear one another. To control for this possibility, mice were always trained in the same pairs.

#### 2.2.3 Video Recording, Processing, and Analysis

Video recordings were triggered by a TTL pulse from an Arduino Nano microcontroller. A separate Arduino generated a frame trigger TTL pulse at 167 Hz, allowing for synchronization of four cameras across two reaching apparatuses. The videos from all four cameras were recorded to a Raspberry pi 4b. The combined videos were separated and converted from mjpeg to avi format using a custom Python script (available ahttps://doi.org/10.5281/zenodo.17290184).

Videos were manually scored by seven blinded scorers to count the number of reaches and abnormal movements produced in each trial. Scorers were blinded to genotype and training day. Videos identified as containing abnormal movements were then re‐viewed by three scorers blinded to genotype and session number. This second round of review was to standardize the presence or absence of abnormal movements across reviewers. Finally, a single blinded reviewer categorized the remaining abnormal movements.

Reaches were defined as a movement of the forelimb towards a sucrose pellet or the pellet wheel. The mean number of reaches that occurred per session was 35.94 (±2.13 SEM). All reach starts are followed by a retrieval (successful pellet grasp and movement of pellet towards mouse) or a miss (either unsuccessful grasp or uncontacted pellet). All retrievals end in either success (pellet consumption) or a fumble (pellet dropped during retrieval).

Reaching performance was quantified by attempt rate (number of trials with at least one reach per session), success rate (number of successful reaches per attempted trials), fumble rate (number of fumbled reaches per retrievals), and abnormal movement rate (number of trials with abnormal movements per session). Because mouse “dystonia” due to torsinA mutations may or may not look like human dystonia, reviewers were instructed to flag any movement they considered unusual for further qualitative review. Well‐described behaviors were sometimes flagged (e.g., grooming); these were removed by the secondary reviewers. Remaining abnormal movements were classified as forelimb or torso twisting, non‐goal directed forelimb movements, or multi‐limb spasming.

Sessions were divided into “early” and “late” for some analyses. Early sessions were those that contained the first 100 reaches. Late sessions were those that contained reaches 401–500. To test whether extended training induces abnormal movements or changes in motor control, some mice were trained past 500 reaches. Behaviors in this extended period were analyzed in 100 reach bins up to 1000 reaches. For each subaction analysis, the first 10 and last 10 reaches were used.

We further subdivided mice into “Learners”/“Non‐Learners” and “High‐Performers”/“Low‐Performers.” Mice were categorized as “Learners” if their success rate increased at all from early (reaches 1–100) to late (reaches 401–500) training. All mice with no change or a decrease in success rate were classified as “Non‐Learners.” Mice were categorized as “High‐Performers” if their success rate in early training (sessions containing reaches 1–100) was above the 50th percentile of early training success rates from all control mice. All mice with success rates at or below that threshold were classified as “Low‐Performers.” The High/Low‐Performer and Learner/Non‐Learner categories were applied independently.

#### 2.2.4 Statistical Analysis

All statistical analyses, unless otherwise specified, were Schrier–Ray–Hare tests. All analyses were performed with MATLAB R2024a, specifically using the function SRH_test (Sinah 2025). KS tests were used to test for normality. Neither control or DYT1‐KI performance distributions were normally distributed, motivating our choice to use non‐parametric tests. Other non‐parametric tests included Fisher's exact test and the Wilcoxon rank sum test, depending on which was contextually appropriate.

## 3 Results

### 3.1 DYT1‐KI Mice Do Not Have Gross Motor Abnormalities During Skilled Reach‐to‐Grasp

We tested DYT1‐KI mice and littermate controls (henceforth control mice) using skilled reach‐to‐grasp to assess motor learning and dexterous motor control (Figure 1A). Each session consisted of 20 reward pellet presentations (“trials”). Each trial lasted 8 s, during which mice could reach for the reward pellet multiple times (35.94 ± 2.13 reaches per session, mean ± SEM). We found no significant difference in success rate (successful reaches per attempted trial, Figure 1B) between DYT1‐KI and control mice (mean success rate, control mice = 16.99% + 2.13% (SEM); mean success rate, DYT1‐KI mice = 18.33% + 3.2% (SEM), Wilcoxon rank sum, *p* = 0.9307) or in the cumulative number of reaches (Figure 1C). These relatively low success rates may be because the pellets rested on top of pedestals (Kernodle et al. 2022), rather than on a shelf where the mouse could slide them part of the way towards their mouth (Phillips et al. 2024). The attempt rate (trials attempted per session, Figure 1D) was higher in DYT1‐KI than in control mice in later sessions. This could reflect increased task‐specific engagement or general hyperactivity as previously described (Dang et al. 2005; Song et al. 2012).

**FIGURE 2:**
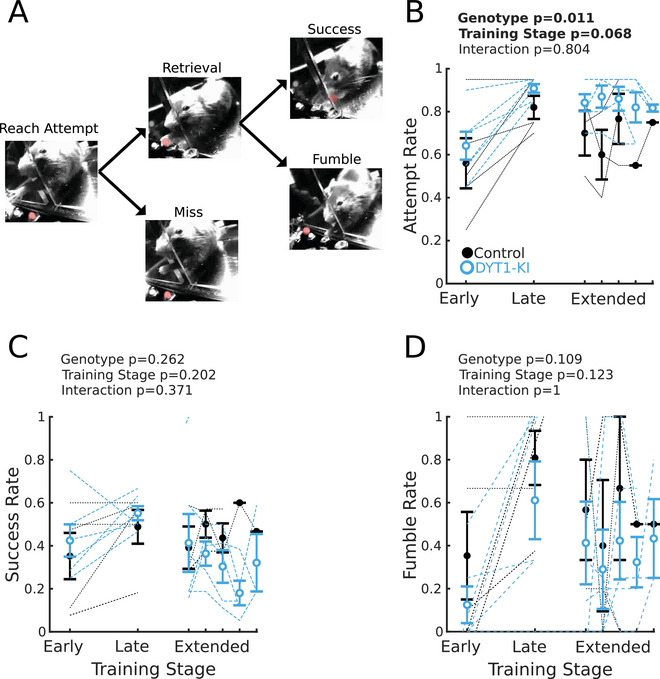
DYT1‐KI mice show no motivational or gross kinematic deficits in skilled reach‐to‐grasp across training stages. (A) Diagram of subaction progression during skilled reach‐to‐grasp. All reach starts are followed by a retrieval (successful pellet grasp and movement of pellet toward mouse) or a miss (either unsuccessful grasp or uncontacted pellet). All retrievals end in either success (pellet consumption) or a fumble (pellet dropped during retrieval). (B) Attempt rate during early (sessions containing reaches 1–100), late (sessions containing reaches 401–500), and extended (sessions after reaching 500) training. (C) Success rate. (D) Fumble rate. For all plots, error bars are ±SEM, DYT1‐KI mice (*n* = 6) are in blue, controls (*n* = 5) in black. Dotted lines represent individual mice. Statistics in bold indicate *p* < 0.05. Full statistics are listed in the supplemental table.

There was also no significant difference in trial outcome between DYT1‐KI and control mice. Individual trials can contain multiple reaches, each of which can end as a miss (no retrieval), a fumble (pellet dropped during retrieval), or a success (Figure 2A). If DYT1‐KI mice develop motor abnormalities as a result of repeating a cortically demanding task, differences between DYT1‐KI and control outcomes should increase with training (early training = sessions containing reaches 1–100, late training = sessions containing reaches 401–500, extended training = sessions containing reaches 501–1000). Attempt rate increased with training (Figure 2B), but there was no effect of training stage specific to DYT1‐KI mice. However, neither success nor fumble rate was affected by genotype or training (Figure 2C,D). Additionally, the only sex difference observed across training stages was for fumble rate, but only in DYT1‐KI mice (Figure S2). In summary, reach‐to‐grasp learning and performance were indistinguishable between DYT1‐KI mice and controls.

### 3.2 DYT1‐KI Mice Do Not Produce a Meaningful Number of Abnormal Movements

**FIGURE 3:**
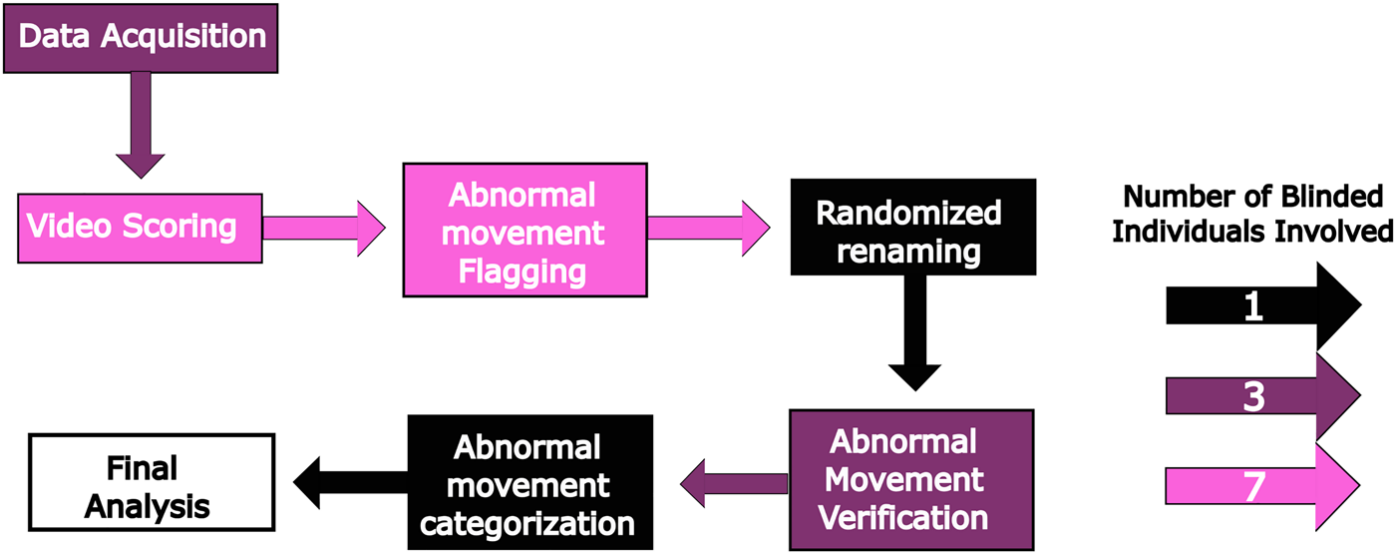
Blinded scoring workflow. Video data was scored for abnormal movements in two rounds. Initially, seven blinded scorers categorized videos as containing an abnormal movement or not. Three blinded scorers then verified that the videos in question contained abnormal movements. Surviving abnormal movements were qualitatively described and grouped based on shared elements by a single final blinded scorer (ATH).

Every video of mice performing skilled reach‐to‐grasp was scored for abnormal movements by scorers blinded to genotype. Scorers were instructed to flag any movement they considered “abnormal” since it is unclear if mouse “dystonia” due to torsinA mutations would directly resemble the human phenotype. After the first round of scoring that included every recorded video, videos flagged as containing abnormal movements were renamed with randomized placeholder names and scored by a second set of blinded reviewers. These scorers provided the final determination of whether movements were indeed abnormal and classified them into three categories (Figure 3; Videos S1–S3). While most mice did not produce any abnormal movements, two DYT1‐KI mice exhibited subtle forelimb shaking prior to reach start in five trials. One control mouse performed a similar forelimb movement in one trial. One DYT1‐KI mouse exhibited brief hindlimb shaking in one trial (this mouse also produced two of the forelimb shaking movements; Table 1). All abnormal movements occurred in late and/or extended training and were qualitatively similar. While we would expect this if overtraining skilled reach‐to‐grasp led to the development of abnormal movements, this increase was seen in both genotypes. We therefore conclude that extended skilled reach‐to‐grasp training does not induce abnormal movements specifically in DYT1‐KI mice.

**TABLE 1:** DYT1‐KI and control mice showed similar varieties of abnormal movements with similar frequencies. While abnormal movements were only seen in a small subset of DYT1‐KI mice, only one trial with an abnormal movement was observed in control mice.

Abnormality	Number of control mice (% of mice)	Number of trials in control mice (% of trials)	Number of DYT1‐KI mice (% of mice)	Number of trials in DYT1‐KI mice (% of trials)
Forepaw flapping	0 (0%)	0 (0%)	2 (33.33%)	4 (0.5435%)
Forepaw pausing	1 (20%)	1 (0.5618%)	0(0%)	0 (0%)
Hindlimb twisting	0 (0%)	0 (0%)	1 (16.67%)	1 (0.2551%)

### 3.3 Subdivision of DYT1‐KI and Control Mice Based on Individual Performance Does Not Uncover Generalizable Behavioral Differences

Because of the incomplete penetrance of DYT1 dystonia, individual mice could develop abnormal movements without a significant group‐level effect. We therefore considered factors that might make individual mice more prone to dystonia‐like movements. Some mice may have experienced more cortical and/or striatal plasticity (Hwang et al. 2019; Luft 2013), which may be reflected in higher (or lower) initial success rates, or greater changes in success rates from early to late training. Mice with an initially higher success rate may not increase their success rate with training, shortening the period during which possible maladaptive plasticity can occur. If motor learning is necessary for the development of abnormal movements, mice with the greatest increase in success (learners) might be more prone to abnormal movements than non‐learners. We therefore divided mice into High and Low performers based on their success rates in the early period (mean number of sessions = 4.8 ± 3.42 STD), and Learners or Non‐Learners based on changes in success rate.

**FIGURE 4:**
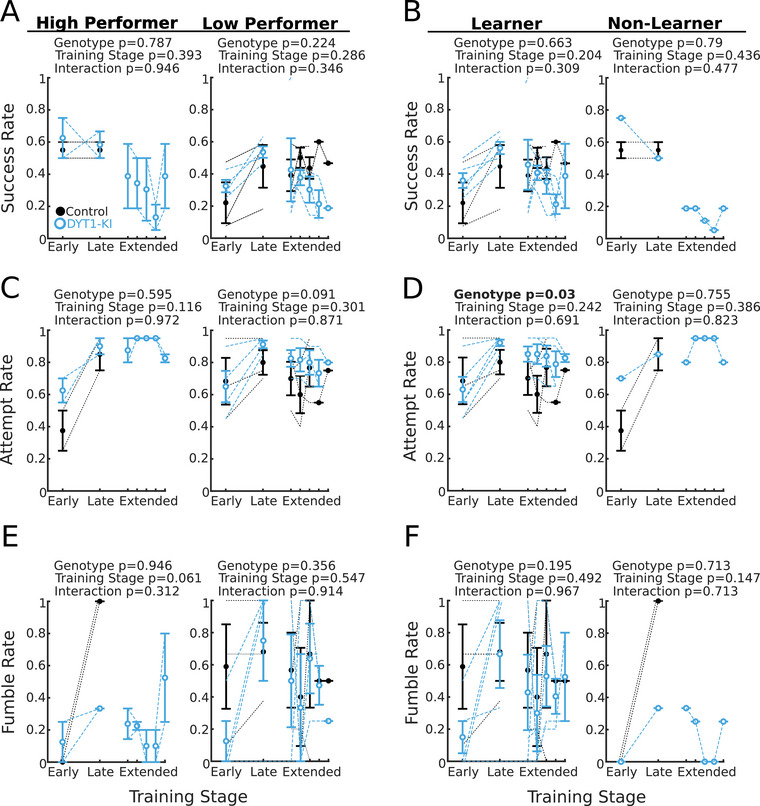
DYT1‐KI mice do not have motor abnormalities that depend on individual performance. (A) Success rates for mice with high and low initial performance. Not all mice produced enough reaches to be included in the extended training section. (B) Success rates for learner and non‐learner mice. (C) Attempt rate for mice with high and low initial performance. (D) Attempt rate for learner and non‐learner mice. (E) Fumble rates for mice with high and low initial performance. (F) Fumble rates for learner and non‐learner mice. Early sessions—sessions containing reaches 1–100; late sessions—sessions containing reaches 400–500; extended sessions—sessions containing reaches over 500. For all plots, error bars are ±SEM, DYT1‐KI mice (*n* = 6) are in blue, controls (*n* = 5) in black. Dotted lines represent individual mice. Statistics in bold indicate *p* < 0.05. Full statistics are listed in the supplemental table.

We found a small but significant difference in success rate (Figure 4A) between high and low performing mice (DYT1‐KI high performing = 2 mice, low performing = 4 mice; control high performing = 2 mice, low performing = 3 mice). However, this was expected as high and low performing mice were defined based on success rate in early training. There was no effect of genotype or training stage on success rate. Attempt rate increased with training, and DYT1‐KI mice attempted to reach more frequently than control mice (Figure 4C). However, there were no significant interactions of genotype, training stage, and/or initial performance. We also found no difference in fumble rate between high and low performing mice (Figure 4E).

No significant difference was found between the success rates (Figure 4B, DYT1‐KI learner = 5 mice, non‐learner = 1 mouse; control learner = 3 mice, non‐learner = 2 mice), attempt rates (Figure 4D), or fumble rates (Figure 4F) of learner and non‐learner mice. There was a significant difference in attempt rate between training stages and between genotypes, but there was no significant interaction of genotype, training stage, or learner/non‐learner categories. We conclude that overtraining skilled reach‐to‐grasp does not facilitate the development of motor abnormalities among functionally‐defined subgroups of DYT1‐KI mice.

### 3.4 Transitions Between Reach Sub‐Actions Are Unaffected by the DYT1‐KI Mutation

**FIGURE 5:**
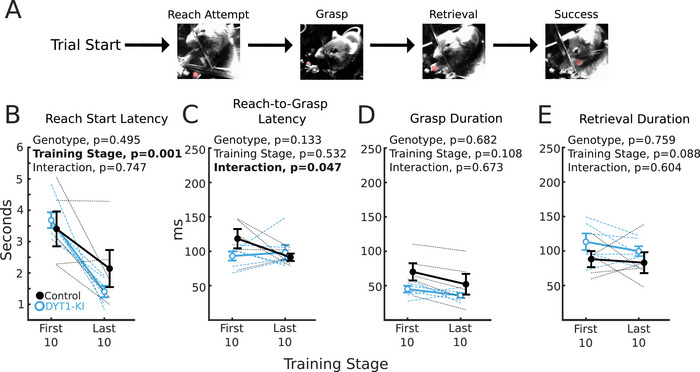
DYT1‐KI and control mice have similar subaction latencies. (A) Diagram of reach subaction progression. (B) Latency from trial start to reach start for DYT1‐KI and control mice in their first 10 reaches and last 10 reaches. (C) Reach‐to‐grasp latency. (D) Duration of pellet grasp. (E) Duration of pellet retrieval. For all plots, error bars are ±SEM, DYT1‐KI mice (*n* = 6) are in blue, controls (*n* = 5) in black. Dotted lines represent individual mice. Statistics in bold indicate *p* < 0.05. Full statistics are listed in the supplemental table.

Gross performance metrics showed no significant difference between DYT1‐KI and control mice, but the DYT1‐KI mutation could cause subtle changes in the timing of reach sub‐actions without affecting reach success (Figure 5A). To determine if DYT1‐KI mice have altered sub‐action timing relative to control mice, we measured the latencies between reach sub‐actions for each mouse's first 10 and last 10 reaches.

Most mice (4/5 controls and 6/6 DYT1‐KI) had shorter latencies between cue‐on and reach start in each mouse's last 10 reaches compared to each mouse's first 10 reaches. There was no difference in the proportion of control or DYT1‐KI mice with latencies that shortened with training (Fisher's exact test, *p* = 0.0769; Figure 5B). We also measured the time elapsed between reach start and grasp start (grasp latency; Figure 5C), full digit extension and paw closure (grasp duration; Figure 5D), and forelimb retraction and the end of the reach (retrieval duration; Figure 5E). We found no significant effect of genotype, training stage, or the interaction of genotype and training stage on any of these intervals. The exception to this was grasp latency—control grasp durations decreased more than DYT1‐KI mice. While we cannot completely rule out a subtle change in reach kinematics, the timing of reach subactions was similar and improved at similar rates in DYT1‐KI mice and controls.

## 4 Discussion

DYT1‐KI mice do not differ significantly from control littermates in how they perform this reach‐to‐grasp task. There was no difference in success rate, fumble rate, or the duration of reach subactions between DYT1‐KI and control mice as a result of training. Contrary to our hypothesis, we observed very few abnormal movements in DYT1‐KI mice after excessive practice. Though DYT1‐KI mice produced slightly more limb‐shaking abnormal movements than control mice, they were in all cases subtle and only present in 2 of 6 mice. Based on our blinded multi‐layered subjective review of the reaching videos, we did not find clear evidence of dystonia‐like (or other) abnormal involuntary movements in DYT1‐KI mice after excessive repetition of reach‐to‐grasp. However, we could have missed subtle changes in forelimb kinematics that might have been detected with modern deep learning methods for behavioral phenotyping (Mathis et al. 2018; Hsu and Yttri 2021; Wiltschko et al. 2015; Hu et al. 2023; Pereira et al. 2019; Pereira et al. 2022; Bova et al. 2020; Bova et al. 2021).

Given that mouse models of human movement disorders often express different movement patterns despite high construct validity (Silvani et al. 2023; Li et al. 2010; Menalled et al. 2009), we were unsure of what motor phenotypes might emerge in DYT1‐KI mice. We therefore used blinded human scoring rather than machine learning approaches to identify potential abnormal movements. Manual scoring limited our sample size, but allowed us to examine large numbers of reaches in each mouse in detail. We therefore cannot be certain that abnormal movements would not have been observed if we had tested more mice for more time, used a different task, or developed a robust unsupervised learning approach to identify abnormal movements. Nonetheless, we are confident that if repetitive reach‐to‐grasp can provoke abnormal movements in adult DYT1‐KI rats, these are at most subtle, rare events. This contrasts with the often dramatic abnormal movements observed in manifesting humans with the ΔGAG mutation.

There are several reasons that repetitive reach‐to‐grasp might not induce abnormal movements in DYT1‐KI mice. The most obvious is that our underlying rationale is wrong, and that over‐training of cortically‐dependent skilled movements is not a “second hit” that partially accounts for the incomplete penetrance in human DYT1 dystonia. Alternatively, overtrained movements could be a factor in human DYT1 dystonia, but murine and human motor systems are too different for DYT1 mice to manifest clear dystonia‐like movements. Primates are more reliant on the motor cortex than rodents for almost all movements. Decorticate rodents can perform a wide array of tasks almost normally, including locomotion and grooming (Travis and Woolsey 1956; Lopes et al. 2023; Berridge and Whishaw 1992). Humans with small motor cortical strokes have significant lasting impairments (Sanchez‐Bezanilla et al. 2021; Feydy et al. 2002). Furthermore, human DYT1 dystonia typically manifests in childhood to adolescence. If years of development are necessary for humans to manifest dystonia, mice may not live long enough to develop plasticity‐mediated dystonia‐like movements. These fundamental differences may create a barrier to using genetic mouse models to replicate human‐like dystonia, at least for torsinA mutations.

Another possible explanation is that we may have missed a critical developmental period. In humans with the Tor1a mutation, dystonia typically manifests around 12 years of age (though some individuals develop or are diagnosed with dystonia later in life). After this critical period, mutation carriers are very unlikely to develop symptomatic dystonia (Li et al. 2021a; Bressman et al. 2000; Semple et al. 2013; Lowe 2024). Critical developmental periods have also been observed in other mouse models. Dlx‐CKO mice have torsinA selectively knocked out of forebrain GABAergic and cholinergic neurons. These mice develop hindlimb clasping when they are 16 days old (Pappas et al. 2015). In an inducible Dlx‐CKO model, suppressing torsinA expression during embryogenesis causes motor and pathologic abnormalities, while suppressing its expression after P70 has no effect. Furthermore, restoring torsinA expression at P21, but not P70, improved the dystonia‐like phenotype (Li et al. 2021b). If DYT1‐KI mice also have a critical period during which motor abnormalities can develop, then exposing adolescent mice to a cortically demanding task may produce abnormal movements. Such an experiment would require a dexterous cortically‐dependent skill that can be learned within days and repeated frequently by juvenile mice.

While DYT1‐KI mice do not exhibit overt dystonia‐like movements, mild motor abnormalities have been described. Multiple studies found increased slips on a narrow balance beam despite normal latencies to traverse the beam (Dang et al. 2005; Yokoi et al. 2011; Yokoi et al. 2015; Wilkes et al. 2023). A murine model expressing human mutant torsinA had decreased latency to fall on the rotarod (Sharma et al. 2005). This was not reproduced in mice expressing mutant murine torsinA (Tanabe et al. 2012), though these mice failed to gain the same performance benefit from cross‐training as controls (Yokoi et al. 2021). This suggests an abnormality in motor circuit plasticity, specifically surrounding motor learning. Why reach‐to‐grasp learning and performance are preserved in DYT1‐KI mice while these gait‐based assessments are impaired is unclear. While the motor cortex is not always necessary for locomotion, it may be utilized enough to stress the cortico‐striatal motor system. This can be seen in situations that require decision‐making or coordination, such as the narrow beam task. Mice with motor cortical lesions are notably impaired in these tasks (Nicholas and Yttri 2024; Cao et al. 2015).

In summary, excessive reach‐to‐grasp training did not induce clear dystonia‐like or other abnormal movements in DYT1‐KI mice. Furthermore, DYT1‐KI learned and performed reach‐to‐grasp at a level similar to controls. This could be because overtraining does not contribute to the human DYT1 manifesting phenotype, innate differences in the human and murine motor systems, or our inability to overtrain mice during a critical developmental period. These results suggest that reach‐to‐grasp is not a useful assay for assessing motor deficits in the murine DYT1‐KI model.

## Author Contributions

**A.T. Hodge**: conceptualization, writing – original draft, writing – review and editing, formal analysis, data curation, methodology, software, investigation. **M. A. Rasheed**: methodology, investigation. **T. Lin**: methodology, investigation. **C. R. Burgess**: conceptualization, funding acquisition, writing – original draft, writing – review and editing, project administration, resources, supervision. **D. K. Leventhal**: conceptualization, funding acquisition, writing – original draft, writing – review and editing, project administration, supervision, resources.

## Funding

This work was supported by NIH grant number 2T32NS007222‐41A1 and NINDS grant number 5R01NS109227‐04.

## Conflicts of Interest

The authors declare no conflicts of interest.

## Supporting information




**Supplementary Figure**: brb371176‐sup‐0001‐FigureS1.pdf

**Supplementary Figure**: brb371176‐sup‐0002‐FigureS2.pdf

**Supplementary Figure**: brb371176‐sup‐0003‐TableS1.pdf

**Supplemental Video 1 ‐ Example Abnormal Movement**. Example video from a DYT1‐KI mouse exhibiting what was classified as an abnormal movement at normal playback speed and 1/10th speed. In this instance, the abnormal movement consisted of tremor‐like repetitive forepaw movement prior to reach initiation (classified as “Forepaw flapping” in Table 1). The red circle highlights the abnormal movement as it occurred.

**Supplemental Video 2 ‐ Example Unsuccessful Reach**. Example video of a control mouse performing an unsuccessful reach without any “abnormal” movements at normal playback speed and 1/10th speed. While unsuccessful, the mouse's reaches were fluid and obviously pellet‐directed.

**Supplemental Video 3 ‐ Example Successful Reach**. Example video of a control mouse performing a successful reach and pellet consumption at normal playback speed and 1/10th speed. As in Video S2, the mouse's reach was fluid and directed toward the pellet. Pellet retrieval was likewise smooth and did not contain any repetitive movements.

**Supplementary Material**: brb371176‐sup‐0007‐SupplementalVideo1.mp4

**Supplementary Material**: brb371176‐sup‐0008‐SupplementalVideo2.mp4

**Supplementary Material**: brb371176‐sup‐0009‐SupplementalVideo3.mp4

## Data Availability

The data that support the findings of this study are openly available in Zenodo at https://zenodo.org/records/17290184, reference number 17290184.
