# Supplementary material for: Cortically Dependent Motor Training Does Not Induce Abnormal Movements in DYT1‐Knock In Mice
Source: Brain Behav. 2025 Dec 31;16(1):e71176. doi: 10.1002/brb3.71176 (PMC12755967; doi:10.1002/brb3.71176)
Supplement: Supplementary file 1 — Supplementary Figure: brb371176‐sup‐0001‐FigureS1.pdf [file BRB3-16-e71176-s003.pdf]

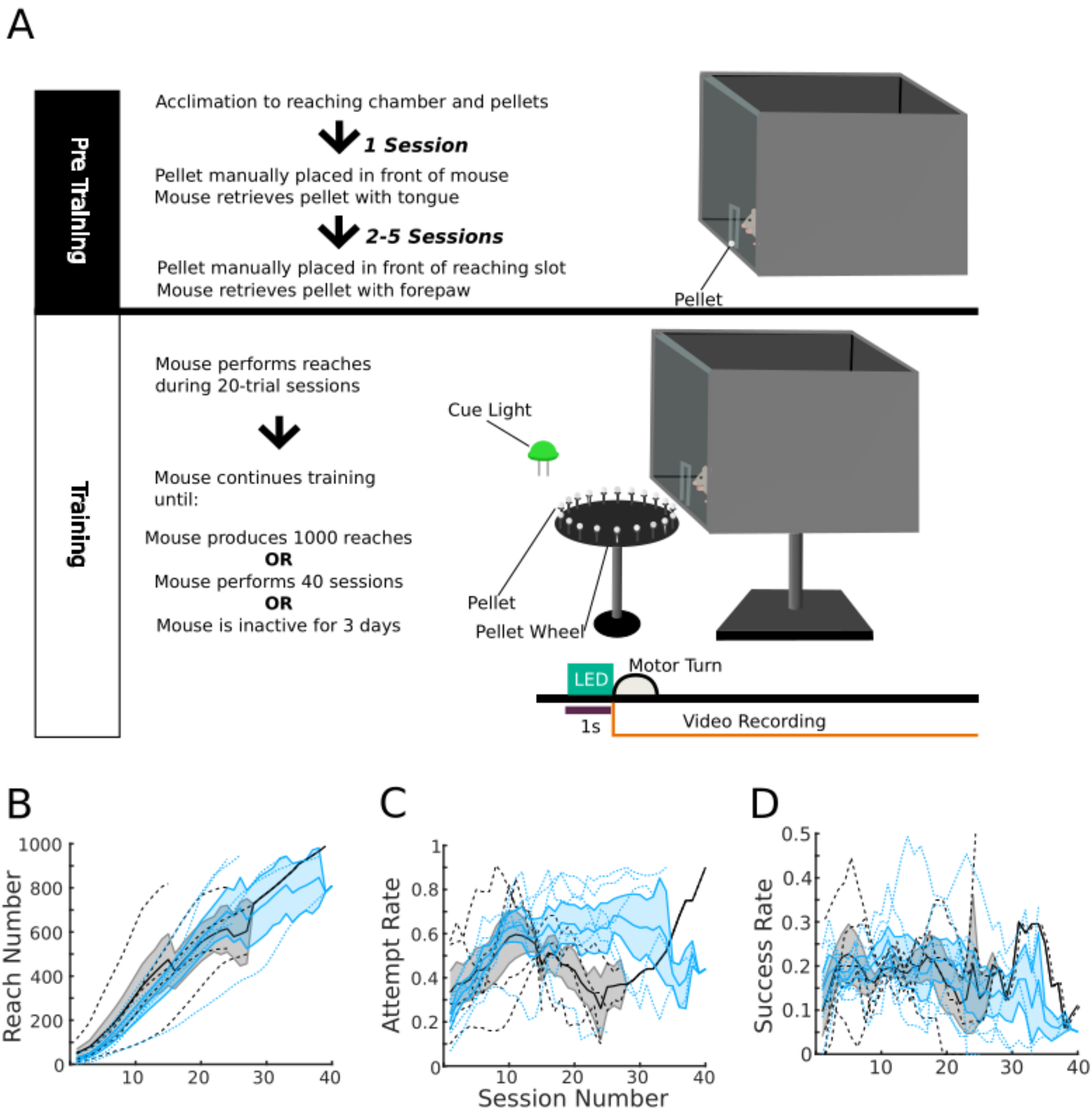
A
Pre Training
Acclimation to reaching chamber and pellets
1 Session
Pellet manually placed in front of mouse
Mouse retrieves pellet with tongue
2-5 Sessions
Pellet manually placed in front of reaching slot
Mouse retrieves pellet with forepaw
Pellet
Training
Mouse performs reaches during 20-trial sessions
Mouse continues training until:
Mouse produces 1000 reaches
OR
Mouse performs 40 sessions
OR
Mouse is inactive for 3 days
Cue Light
Pellet
Pellet Wheel
LED
Motor Turn
1s
Video Recording
B
Reach Number
C
Attempt Rate
Session Number
D
Success Rate

Supplemental Figure 1 Skilled reach-to-grasp training schedule. A. Pre-training and Training schedule. Mice were acclimated to the reaching chamber and sucrose pellets for one 20-minute session. After this acclimation period, sucrose pellets were only available through the central reaching slot in the front of the reaching chamber. Initially, pellets were placed directly in the slot to allow mice to consume pellets without reaching. Once mice searched for pellets in the reaching slot, pellets were placed just outside of the reaching chamber. When mice readily reached for pellets outside of the reaching chamber for 2 consecutive sessions, pre-training stopped and training started. Training continued until mice either produced 1000 reaches, performed 40 sessions, or became fully inactive for 3 consecutive days. **B.** Cumulative reach number for DYT1-KI and Control mice for 40 sessions. **C.** Attempt Rate for DYT1-KI and Control mice for 40 sessions. **D.** Success Rate for DYT1-KI and Control mice for 40 sessions. In panels **B** - **D**, DYT1-KI mice are represented by blue lines while Control mice are represented by black lines.
