# Supplementary material for: Cortically Dependent Motor Training Does Not Induce Abnormal Movements in DYT1‐Knock In Mice
Source: Brain Behav. 2025 Dec 31;16(1):e71176. doi: 10.1002/brb3.71176 (PMC12755967; doi:10.1002/brb3.71176)
Supplement: Supplementary file 2 — Supplementary Figure: brb371176‐sup‐0002‐FigureS2.pdf [file BRB3-16-e71176-s005.pdf]

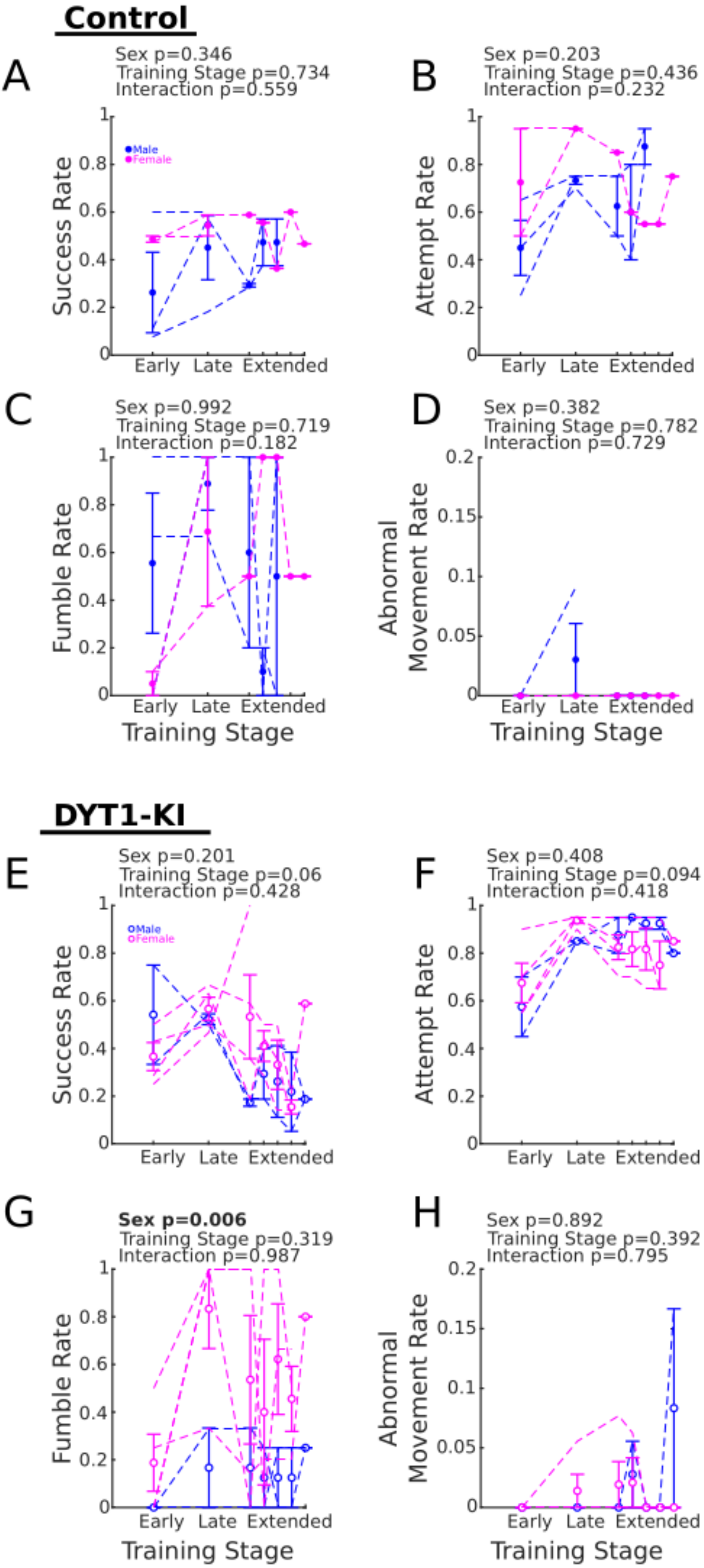
Control
A
Sex p=0.346
Training Stage p=0.734
Interaction p=0.559
Success Rate
Male
Female
Early Late Extended
B
Sex p=0.203
Training Stage p=0.436
Interaction p=0.232
Attempt Rate
Early Late Extended
C
Sex p=0.992
Training Stage p=0.719
Interaction p=0.182
Fumble Rate
Early Late Extended
Training Stage
D
Sex p=0.382
Training Stage p=0.782
Interaction p=0.729
Abnormal Movement Rate
Early Late Extended
Training Stage
DYT1-KI
E
Sex p=0.201
Training Stage p=0.06
Interaction p=0.428
Success Rate
Male
Female
Early Late Extended
F
Sex p=0.408
Training Stage p=0.094
Interaction p=0.418
Attempt Rate
Early Late Extended
G
Sex p=0.006
Training Stage p=0.319
Interaction p=0.987
Fumble Rate
Early Late Extended
Training Stage
H
Sex p=0.892
Training Stage p=0.392
Interaction p=0.795
Abnormal Movement Rate
Early Late Extended
Training Stage

Supplemental Figure 2 Sex effects on the development of abnormal movements in DYT1-KI and Control mice. A. Success rates for male and female Control mice in early, late, and extended training. There was no significant interaction of sex and training stage (SRH test H=2.996, *p=0.559*). There was no individual effect of sex (SRH test H=0.89, *p=0.346*) or training category (SRH test H=3.57, *p=0.734*). **B.** Attempt rates for male and female Control mice in early, late, and extended training. There was no significant interaction of sex and training stage (SRH test H=5.59, *p=0.232*). There was no individual effect of sex (SRH test H=1.62, *p=0.203*) or training category (SRH test H=5.88, *p=0.436*). **C.** Fumble rates for male and female Control mice in early, late, and extended training. There was no significant interaction of sex and training stage (SRH test H=6.23, *p=0.182*). There was no individual effect of sex (SRH test H=0.00011, *p=0.992*) or training category (SRH test H=3.69, *p=0.719*). **D.** Abnormal movement rates for male and female Control mice in early, late, and extended training. There was no significant interaction of sex and training stage (SRH Test H=2.04, *p=0.73*). There was no individual effect of sex (SRH test H=0.764, *p=0.382*) or training category (SRH test H=3.21, *p=0.782*). **E.** Success rates for male and female DYT1-KI mice in early, late, and extended training. There was no significant interaction of sex and training stage (SRH test H=5.96, *p=0.43*). There was no individual effect of sex (SRH test H=1.64, *p=0.2*) or training category (SRH test H=12.1, *p=0.06*). **F.** Attempt rates for male and female DYT1-KI mice in early, late, and extended training. There was no significant interaction of sex and training stage (SRH test H=6.04, *p=0.42*). There was no individual effect of sex (SRH test H=0.68, *p=0.408*) or training category (SRH test H=10.83, *p=0.094*). **G.** Fumble rates for male and female DYT1-KI mice in early, late, and extended training. There was no significant interaction of sex and training stage (SRH test H=0.97, *p=0.99*). There was an individual effect of sex (SRH test H=7.53, *p=0.006*) but not training category (SRH test H=7.02, *p=0.32*). **H.** Abnormal movement rates for male and female DYT1-KI mice in early, late, and extended training. There was no significant interaction of sex and training stage (SRH test H=3.11, *p=0.795*). There was no individual effect of sex (SRH test H=0.018, *p=0.89*) or training category (SRH test H=6.29, *p=0.392*). In all panels, males are represented by blue lines and dots while females are represented by pink lines and dots.
