# Supplementary material for: Cortically Dependent Motor Training Does Not Induce Abnormal Movements in DYT1‐Knock In Mice
Source: Brain Behav. 2025 Dec 31;16(1):e71176. doi: 10.1002/brb3.71176 (PMC12755967; doi:10.1002/brb3.71176)
Supplement: Supplementary file 3 — Supplementary Figure: brb371176‐sup‐0003‐TableS1.pdf [file BRB3-16-e71176-s001.pdf]

Figure 1				
**Panel B**	Sum of Squares	DF	H statistic	P value
Genotype	4260.3	1	0.80843	0.36859
Session Number	1.84E+05	24	34.826	0.07102
Interaction	1.03E+05	24	19.525	0.72342
**Panel C**	Sum of Squares	DF	H statistic	P value
Genotype	291.4	1	0.55307	0.81407
Session Number	2.25E+05	24	42.702	0.0108
Interaction	1.04E+05	24	19.74	0.7144
**Panel D**	Sum of Squares	DF	H statistic	P value
Genotype	25893	1	4.7741	0.02889
Session Number	2.63E+05	24	48.421	0.002238
Interaction	1.00E+05	24	18.437	0.78133
Figure 2				
**Panel B**	Sum of Squares	DF	H statistic	P value
Genotype	1748.3	1	6.522	0.010655
Training Stage	3.14E+03	6	11.729	0.068292
Interaction	8.14E+02	6	3.0367	0.80422
**Panel C**	Sum of Squares	DF	H statistic	P value
Genotype	345.68	1	1.259	0.26184
Training Stage	2.34E+03	6	8.5276	0.20194
Interaction	1.78E+03	6	6.484	0.37121
**Panel D**	Sum of Squares	DF	H statistic	P value
Genotype	680.23	1	2.5698	0.10892
Training Stage	2.65E+03	6	10.028	0.12346
Interaction	4.48E+01	6	0.16916	0.99991

Figure 4				
**Panel A High Performer**	Sum of Squares	DF	H statistic	P value
Genotype	2	1	0.073276	0.78663
Training Stage	1.71E+02	6	6.2775	0.39283
Interaction	1.25E-01	1	0.0045797	0.94605
**Panel A Low Performer**	Sum of Squares	DF	H statistic	P value
Genotype	192.3	1	1.4813	0.22357
Training Stage	9.60E+02	6	7.3914	0.28616
Interaction	8.74E+02	6	6.7329	0.34626
**Panel A Pooled**	Sum of Squares	DF	H statistic	P value
Genotype	249.72	1	0.90954	0.34024
Training Stage	2052.2	6	7.4745	0.27918
Initial Performance	**1073.3**	**1**	**3.9093**	**0.04802**
Genotype x Training Stage	1714.7	6	6.2451	0.3963
Genotype x Initial Performance	44.531	1	0.16219	0.68715
Training Stage x Initial Performance	1084.5	6	3.9499	0.68346
Interaction	0.00054825	1	2.00E-06	0.68346
**Panel B Learner**	Sum of Squares	DF	H statistic	P value
Genotype	34.225	1	0.19035	0.66263
Training Stage	1.53E+03	6	8.4892	0.20441
Interaction	1.28E+03	6	7.1291	0.30907
**Panel B**	Sum of Squares	DF	H statistic	P value

**Non-Learner**				
Genotype	0.75	1	0.07109	0.78976
Training Stage	6.21E+01	6	5.8858	0.4361
Interaction	5.33E+00	1	0.50553	0.47708
**Panel B Pooled**	Sum of Squares	DF	H statistic	P value
Genotype	23.885	1	0.086992	0.76804
Training Stage	1957.8	6	7.1308	0.30892
Initial Performance	4.5188	1	0.016458	0.89792
Genotype x Training Stage	1887.4	6	6.8741	0.33265
Genotype x Initial Performance	74.449	1	0.27116	0.60256
Training Stage x Initial Performance	1822.6	6	6.6382	0.35561
Interaction	69.955	1	0.25479	0.61372
**Panel C High Performer**	Sum of Squares	DF	H statistic	P value
Genotype	7.0312	1	0.28225	0.59523
Training Stage	2.54E+02	6	10.213	0.11595
Interaction	3.13E-02	1	0.0012544	0.97175
**Panel C Low Performer**	Sum of Squares	DF	H statistic	P value
Genotype	366.35	1	2.8542	0.091136
Training Stage	9.26E+02	6	7.2154	0.30138
Interaction	3.18E+02	6	2.4741	0.87135
**Panel C Pooled**	Sum of Squares	DF	H statistic	P value
Genotype	734.87	1	2.7414	0.097779

Training Stage	3166.5	6	11.812	0.066287
Initial Performance	411.27	1	1.5342	0.21548
Genotype x Training Stage	471.99	6	1.7607	0.94034
Genotype x Initial Performance	17.961	1	0.067003	0.79575
Training Stage x Initial Performance	1262.1	6	4.7081	0.58175
Interaction	153.42	1	0.57234	0.44933
**Panel D Learner**	Sum of Squares	DF	H statistic	P value
Genotype	830.41	1	4.7171	0.029865
Training Stage	1.40E+03	6	7.95	0.24179
Interaction	6.86E+02	6	3.8941	0.69101
**Panel D Non-Learner**	Sum of Squares	DF	H statistic	P value
Genotype	1.0208	1	0.097687	0.75462
Training Stage	6.63E+01	6	6.3403	0.38617
Interaction	5.21E-01	1	0.049841	0.82334
**Panel D Pooled**	Sum of Squares	DF	H statistic	P value
Genotype	**1265.5**	**1**	**4.7209**	**0.029798**
Training Stage	3131.5	6	11.682	0.069452
Initial Performance	9.3197	1	0.034767	0.85208
Genotype x Training Stage	733.65	6	2.7369	0.84108
Genotype x Initial Performance	6.9509	1	0.02593	0.87207

Training Stage x Initial Performance	735.48	6	2.7437	0.84026
Interaction	342.43	1	1.2774	0.25838
**Panel E High Performer**	Sum of Squares	DF	H statistic	P value
Genotype	0.125	1	0.0045406	0.94628
Training Stage	3.31E+02	6	12.023	0.061461
Interaction	2.81E+01	1	1.0216	0.31213
**Panel E Low Performer**	Sum of Squares	DF	H statistic	P value
Genotype	104.56	1	0.85303	0.3557
Training Stage	6.10E+02	6	4.9785	0.54657
Interaction	2.53E+02	6	2.0618	0.91393
**Panel E Pooled**	Sum of Squares	DF	H statistic	P value
Genotype	391.08	1	1.4774	0.22418
Training Stage	2714.4	6	10.254	0.11434
Initial Performance	669.93	1	2.5308	0.11164
Genotype x Training Stage	210.09	6	0.79368	0.99224
Genotype x Initial Performance	32.64	1	0.12331	0.72547
Training Stage x Initial Performance	959.93	6	3.6264	0.72708
Interaction	732.64	1	2.7678	0.096181
**Panel F Learner**	Sum of Squares	DF	H statistic	P value
Genotype	291.62	1	1.6796	0.19497
Training Stage	9.39E+02	6	5.4104	0.49235

Interaction	2.39E+02	6	1.3792	0.96711
**Panel F Non-Learner**	Sum of Squares	DF	H statistic	P value
Genotype	1.333	1	0.13536	0.71293
Training Stage	9.36E+01	6	9.5057	0.14707
Interaction	1.33E+00	1	0.13536	0.71293
**Panel F Pooled**	Sum of Squares	DF	H statistic	P value
Genotype	612.04	1	2.3122	0.12837
Training Stage	2696.7	6	10.188	0.11697
Initial Performance	661.91	1	2.5005	0.11381
Genotype x Training Stage	173.29	6	0.65465	0.99542
Genotype x Initial Performance	0.3347	1	0.0012644	0.97163
Training Stage x Initial Performance	1123.8	6	4.2454	0.6435
Interaction	307.57	1	4.2454	0.6435
Figure 5				
**Panel B**	Sum of Squares	DF	H statistic	P value
Genotype	19.643	1	0.46584	0.49491
Session Number	4.64E+02	1	10.996	0.00091289
Interaction	4.39E+00	1	0.1041	0.74696
**Panel C**	Sum of Squares	DF	H statistic	P value
Genotype	95.071	1	2.2598	0.13277
Session Number	1.64E+01	1	0.39003	0.53228
Interaction	1.66E+02	1	3.9512	0.046837
**Panel D**	Sum of Squares	DF	H statistic	P value

Genotype	7.0714	1	0.1677	0.68216
Session Number	1.09E+02	1	2.5882	0.10766
Interaction	7.51E+00	1	0.17802	0.67308
**Panel E**	Sum of Squares	DF	H statistic	P value
Genotype	3.9777	1	0.094386	0.75867
Session Number	1.23E+02	1	2.9165	0.087679
Interaction	1.13E+01	1	0.26858	0.60429

Supplemental table - Extended Statistics for Figures 1, 2, 4, and 5. All results in this table are from Schrier-Ray-Hare tests
