# Supplementary material for: Cortically Dependent Motor Training Does Not Induce Abnormal Movements in DYT1‐Knock In Mice
Source: Brain Behav. 2025 Dec 31;16(1):e71176. doi: 10.1002/brb3.71176 (PMC12755967; doi:10.1002/brb3.71176)
Supplement: Supplementary file 4 — Supplemental Video 1 ‐ Example Abnormal Movement. Example video from a DYT1‐KI mouse exhibiting what was classified as an abnormal movement at normal playback speed and 1/10th speed. In this instance, the abnormal movement consisted of tremor‐like repetitive forepaw movement prior to reach initiation (classified as “Forepaw flapping” in Table 1). The red circle highlights the abnormal movement as it occurred. [file BRB3-16-e71176-s007.pdf]

Supplemental Video 1 - Example Abnormal Movement. Example video from a DYT1-KI mouse exhibiting what was classified as an abnormal movement at normal playback speed and 1/10th speed. In this instance, the abnormal movement consisted of tremor-like repetitive forepaw movement prior to reach initiation (classified as “Forepaw flapping” in Table 1). The red circle highlights the abnormal movement as it occurred.
