# Supplementary material for: Cortically Dependent Motor Training Does Not Induce Abnormal Movements in DYT1‐Knock In Mice
Source: Brain Behav. 2025 Dec 31;16(1):e71176. doi: 10.1002/brb3.71176 (PMC12755967; doi:10.1002/brb3.71176)
Supplement: Supplementary file 5 — Supplemental Video 2 ‐ Example Unsuccessful Reach. Example video of a control mouse performing an unsuccessful reach without any “abnormal” movements at normal playback speed and 1/10th speed. While unsuccessful, the mouse's reaches were fluid and obviously pellet‐directed. [file BRB3-16-e71176-s009.pdf]

Supplemental Video 2 - Example Unsuccessful Reach. Example video of a Control mouse performing an unsuccessful reach without any “abnormal” movements at normal playback speed and 1/10th speed. While unsuccessful, the mouse’s reaches were fluid and obviously pellet-directed.
