# Supplementary material for: Cortically Dependent Motor Training Does Not Induce Abnormal Movements in DYT1‐Knock In Mice
Source: Brain Behav. 2025 Dec 31;16(1):e71176. doi: 10.1002/brb3.71176 (PMC12755967; doi:10.1002/brb3.71176)
Supplement: Supplementary file 6 — Supplemental Video 3 ‐ Example Successful Reach. Example video of a control mouse performing a successful reach and pellet consumption at normal playback speed and 1/10th speed. As in Video S2, the mouse's reach was fluid and directed toward the pellet. Pellet retrieval was likewise smooth and did not contain any repetitive movements. [file BRB3-16-e71176-s002.pdf]

Supplemental Video 3 - Example Successful Reach. Example video of a Control mouse performing a successful reach and pellet consumption at normal playback speed and 1/10th speed. As in Supplemental Video 2, the mouse's reach was fluid and directed towards the pellet. Pellet retrieval was likewise smooth and did not contain any repetitive movements.
